# Differential Sialylation of Serpin A1 in the Early Diagnosis of Parkinson’s Disease Dementia

**DOI:** 10.1371/journal.pone.0048783

**Published:** 2012-11-08

**Authors:** Sarah Jesse, Stefan Lehnert, Olaf Jahn, Lucilla Parnetti, Hilkka Soininen, Sanna-Kaisa Herukka, Petra Steinacker, Saskia Tawfik, Hayrettin Tumani, Christine A. F. von Arnim, Manuela Neumann, Hans A. Kretzschmar, Hasan Kulaksiz, Martin Lenter, Jens Wiltfang, Boris Ferger, Bastian Hengerer, Markus Otto

**Affiliations:** 1 Department of Neurology, University of Ulm, Ulm, Germany; 2 Proteomics Group, Max-Planck-Institute for Experimental Medicine, Goettingen, Germany; 3 DFG Research Center for Molecular Physiology of the Brain, Goettingen, Germany; 4 Department of Neurology, University of Perugia, Perugia, Italy; 5 Department of Neurology, University of Eastern Finland and Kuopio University Hospital, Kuopio, Finland; 6 Institute of Neuropathology, University of Zurich, Zurich, Switzerland; 7 Institute of Neuropathology, Ludwig Maximilians University, Munich, Germany; 8 Department of Internal Medicine, University of Ulm, Ulm, Germany; 9 CNS Diseases Research, Boehringer Ingelheim GmbH & Co. KG, Biberach an der Riss, Germany; 10 Department of Psychiatry, University of Essen-Duisburg, Essen-Duisburg, Germany; Mayo Clinic, United States of America

## Abstract

The prevalence of Parkinson’s disease (PD) increases with age. Up to 50% of PD show cognitive decline in terms of a mild cognitive impairment already in early stages that predict the development of dementia, which can occur in up to 80% of PD patients over the long term, called Parkinson’s disease dementia (PDD). So far, diagnosis of PD/PDD is made according to clinical and neuropsychological examinations while laboratory data is only used for exclusion of other diseases. The aim of this study was the identification of possible biomarkers in cerebrospinal fluid (CSF) of PD, PDD and controls (CON) which predict the development of dementia in PD. For this, a proteomic approach optimized for CSF was performed using 18 clinically well characterized patients in a first step with subsequent validation using 84 patients. Here, we detected differentially sialylated isoforms of Serpin A1 as marker for differentiation of PD versus PDD in CSF. Performing 2D-immunoblots, all PDD patients could be identified correctly (sensitivity 100%). Ten out of 24 PD patients showed Serpin A1 isoforms in a similar pattern like PDD, indicating a specificity of 58% for the test-procedure. In control samples, no additional isoform was detected. On the basis of these results, we conclude that differentially sialylated products of Serpin A1 are an interesting biomarker to indicate the development of a dementia during the course of PD.

## Introduction

An increasing prevalence for Parkinson’s disease (PD) can be detected in advanced age, with 1% among 60-year-olds and 3% in the 80-year-old age-group [1]. Of note is that patients with PD have a roughly 6-times higher risk to develop a dementia than an age-matched healthy control group [2]. Up to 50% of PD show cognitive decline in terms of a mild cognitive impairment already in early stages that predicts the development of dementia, which can occur in up to 80% of PD patients over the long term [3], [4]. The dementia syndrome usually develops after approximately 8 to 10 years and has a strong influence not only on the course of the disease but also on the social environment with higher requirements for families and caretakers during everyday life. The latter causes a psychological strain for the patient and family [5], leading to increased stress during home care [6] with growing need for professional care. The dementia syndrome is also accompanied with a worse prognosis as regards disease-progression and life expectancy [7]. Early treatment is critical for the modification of the disease progress as acetylcholine esterase inhibitors have only a delaying effect on worsening of cognitive deficits in early stages when neurodegeneration is not exessively advanced. [8]. Therefore, there is a clear need for a biomarker to define patients at risk.

Neuropathologically, PDD is characterized by cortical Lewy bodies that also occur in patients with dementia with Lewy bodies. However it is heretofore unclear whether both diseases are a matter of a single one. By definition, diagnosis of PDD is made when the onset of dementia is more than one year after the onset of Parkinsonism whereas DLB should be diagnosed when dementia occurs before or concurrently with Parkinsonism [9], [10], [11], [12], [13]. As a rule both PDD and DLB are associated with histological changes of Alzheimer's disease [14]. It has been shown that Lewy bodies contain alpha-synuclein, a presynaptic filament protein that mainly is expressed in the terminal endings of neurons. Therefore, an obvious working theory is that these Lewy bodies are directly linked to the pathophysiological processes, especially that alpha-synuclein inclusions are mostly present in surviving cells and less so in apoptotic cells, suggesting that these inclusions may play a protective role in cell death by sequestering toxic molecular species [15], [16]. Regarding the formation of alpha-synuclein containing inclusion bodies and their importance in neuropathological alterations, Braak et al. were able to indicate a topographical extent of these lesions with an initial onset in the dorsal IX/X-motor nucleus and the intermediate reticular zone in the brain stem, proceeding with an ascending course to cortical structures, beginning with the anteromedial temporal mesocortex [17], [18], [19]. As a possible link between neurotoxicity, aggregation and propagation it might be concluded that species of neurotoxic oligomers can be transformed to oligomers which are not neurotoxic, but have a higher tendency of further aggregation [20], [21].

We and others made attempts to improve the early diagnosis of dementia in PD patients by measurement of alpha-synuclein or proposed alpha-synuclein aggregates and by known biomarkers in CSF and serum [22], [23], [24], [25]. However, for prognosis of disease progression in an individual patient this neurochemical profile is currently of limited use [22].

Using an optimized protocol for the proteomic analysis of CSF, which particularly accounts for the brain protein variation caused by CSF flow [26], we investigated a set of well defined clinical groups of patients with PD, PDD and a control group to find a marker which can differentiate between the demented and non-demented persons. Thereby, we found that PDD patients can be identified on the basis of differentially sialylated isoforms of Serpin A1 in CSF. In a second step, this protein was validated in an independent set of patients and investigated in human brain material.

## Results

### PDD Patients can be Identified on the Basis of Serpin A1 Isoforms

**Figure 1:**
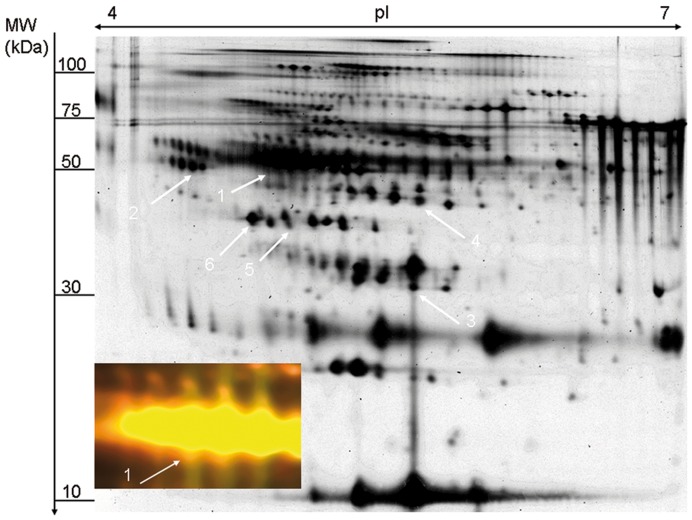
Representative 2D-DIGE gel of CyDye-labeled CSF-proteins. Indication is given in black-white manner for better visibility. Arrows indicate identified protein spots: number 1 for Serpin A1, number 2 for Fetuin A, number 3 for Ceruloplasmin, number 4 for Serpin F1, number 5 for Haptoglobin and number 6 for Zinc-alpha-2 glycoprotein (numbers correlate with the numbers in the first row of Table 2). The magnification shows spot number 1 in CyDye overlay. Abbreviations: pI = isoelectric point of the proteins; MW  =  molecular weight in kilo-Dalton.

In the first step of our study, identification of regulated proteins relevant for differentiation of PD versus PDD was approached by means of 2D-DIGE experiments. CSF samples of 6 patients per group (PD, PDD, CON) were analysed, whereby an internal standard consisting of a mixture of all 18 samples was used to ensure the comparability of the gels during the subsequent software-based evaluation. No pooling was performed, but two samples from patients of different groups were loaded on a gel together with the internal standard so that 18 gels were analysed in total. Also a dye-switch was made to exclude false results due to preferential binding of proteins to one dye. A representative gel is shown in Figure 1. Relevant proteins were identified using MALDI-ToF MS/MS analysis. Characteristics of all patients are given in Table 1; Spot data for the identified proteins are shown in Table 2.

**Table 1:** Relevant parameters of all groups investigated.

Disease	N	m/f	Age	Tau (pg/ml)	MMST	Hoehn&Yahr
PD	24	9/15	66±9	264±140	26±4	2.1±1.1
PDD	21	10/11	76±8	326±250	20±6	2.9±1.1
AD	9	5/4	70±13	786±427	23±4	–
FTLD	6	4/2	64±7	450±387	27±2	–
CON	24	9/15	70±06	–	–	–

Data are indicated as mean±SD.

Abbreviations: PD  =  Parkinson’s disease, PDD  =  Parkinson’s dementia, AD  =  Alzheimer’s disease, FTLD  =  frontotemporal lobar degeneration, CON  =  control persons, m/f  =  male/female, MMST  =  minimal mental status test.

**Table 2:** 2D-DIGE analysis and identification of selected CSF proteins.

Spot	Protein name	Regulation	Ratio	Swiss-Prot accession	MW [kDa]	pI	PMF coverage [%]	PMFscore^a)^	Peptidesequenced	MS/MS ionscore^b)^
1	Alpha-1-antitrypsin(Serpin A1)	PDD vs. CONPDD vs. PD	1.61 1.80	P01009	46878	5.4	55	257	LYHSEAFTVNFGDTEEAKLQHLENELTHDIITKTDTSHHDQDHPTFNKLYHSEAFTVNFGDTEEAKK	93844235
2	Alpha-2-HSglycoprotein(Fetuin A)	CON vs. PDD	−1.32	P02765	40098	5.4	22	63	EHAVEGDCDFQLLKHTFMGVVSLGSPSGEVSHPHTFMGVVSLGSPSGEVSHPR	1123825
3	Ceruloplasmin	PD vs. CON	−2.23	P00450	122983	5.4	23	122	GAYPLSIEPIGVR DLYSGLIGPLIVCR	4633
4	Pigment epithelium-derived factor(Serpin F1)	PDD vs. CON	1.59	P36955	46484	6.0	42	144	TSLEDFYLDEER LAAAVSNFGYDLYR	3825
5	Haptoglobin	PDD vs. CON	1.75	P00738	45861	6.1	35	115	YVMLPVADQDQCIR VGYVSGWGR	5836
6	Zinc-alpha-2 glycoprotein	CON vs. PDD	−1.79	P25311	34079	5.5	54	214	AREDIFMETLK AYLEEECPATLRYSLTYIYTGLSK	54

Abbreviations: PD  =  Parkinson’ disease, PDD  =  Parkinson’s dementia, CON  =  control persons, pI  =  isoelectric point of the proteins, PMF  =  peptide mass fingerprint, MS  =  mass spectrometry.

a) Mascot protein score obtained for the peptide mass fingerprint (PMF). The significance threshold was 56.

b) Mascot MS/MS ion scores obtained for the individual peptides sequenced. The significance threshold was 17–29 depending on how many peptides fell within the mass tolerance window about the precursor mass. Only the top ranking peptides matching a query for the first time (“bold red hits”) are reported.

**Figure 2:**
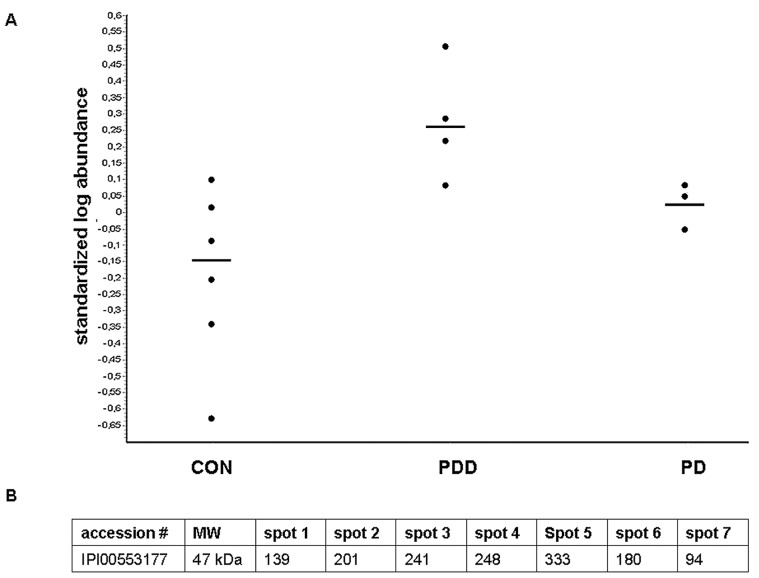
Identification and regulation of Serpin A1 and its different isoforms. **2A** illustrates the 2D-DIGE analysis with the pixel volume distribution for Serpin A1 corresponding to number 2 in Figure 1. The horizontal lines indicate the median value. Average ratios CON vs PDD 2.34, PDD vs PD 1.80. p-values CON vs PDD 0.014, PDD vs PD 0.043. **2B** shows the isoform distribution of Serpin A1 of a representative PDD gel with spectral counts for the respective isoforms.

**Figure 3:**
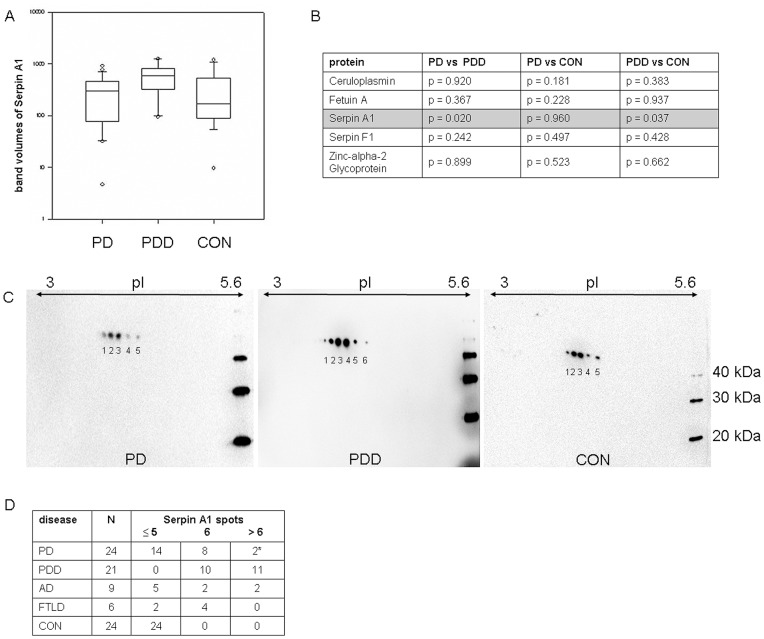
1D- and 2D-immunoblots of Serpin A1. **3A** shows 1D-immunoblot band volumes (adjusted for membrane background) of Serpin A1. **3B** shows the statistical analysis for the 1D-immunoblot validation of all proteins found to be regulated in the 2D DIGE experiment. Only Serpin A1 displayed a significant regulation. **3C** illustrates the 2D immunoblot of Serpin A1 with the different spot-pattern in PD/CON and PDD with the relevant additional spots 1 and/or 2 in PDD. **3D** shows the distribution of spot pattern in the different groups (PDD, PD versus the dementia subgroups and CON). *Both PD-patients with 7/9 Serpin A1 spots developed dementia in the course of the disease. Abbreviations: PD  =  Parkinson’s disease, PDD  =  Parkinson’s dementia, CON  =  control persons, AD  =  Alzheimer’s disease, FTLD  =  frontotemporal lobar degeneration, pI  =  isoelectric point of the proteins.

In a second step, we examined the reproducibility of the 2D-DIGE-data using 1D-immunoblotting as complementary approach. In order to maintain comparability with the proteomic 2D-DIGE, samples were also used volume-normalized. After quantitative analysis of the protein-bands, Serpin A1 showed a statistically significant regulation between PDD on one side and PD/CON on the other (Figure 2A) with large overlap between the analysed groups (Figure 3A/B).

On the basis of the pixel volumes out of the DIGE experiments, we had expected a more prominent difference of Serpin A1 regulation in the subsequent validation phase as seen in our 1D-immunoblot data. As the most likely explanation for this discrepancy was that the Serpin A1 regulation seen in 2D-DIGE was related to particular isoforms (which are not separated in the conventional 1D-immunoblotting method), we performed 2D-immunoblots to test for the presence of differential Serpin A1 isoforms in the groups. Here indeed, a different isoform-pattern was detected with usually ≤5 spots in PD and CON and 6 or more spots in PDD. Spots indicated as spot 1 and spot 2 are additionally seen in PDD patients (Figure 3C). These results could also be reproduced in the CSF-samples from Kuopio/Finland and Perugia/Italy, which were investigated in a blinded manner to test reproducibility of our data and to exclude a centre effect caused by preanalytical handling procedures of CSF-samples.

In a next step, we were interested in the sensitivity and specificity of Serpin A1 regarding its relevance as a possible diagnostic marker to differentiate between PD and PDD. For this, we analysed the cut-off of 5.5 spots obtained by ROC analysis and also iterative testing. Using this cut-off (or ≥6 spots), we compared PD and PDD and found a specificity of 58% and a sensitivity of 100% by 2D immunoblot approach. In the relevant diagnostic PD group the additional spots were seen in 10 out of 24 patients; interestingly, two patients who presented with more than 6 spots developed a dementia in the course of disease (one patient developed dementia already after one year whereas the other one remained stable over a longer time). To test specificity among dementia subgroups, a small set of patients with other dementia like Alzheimer’s disease (AD) and fronto-temporal lobar degeneration (FTLD) were analyzed whereby the specificity in the subgroups ranged from 71% in AD to 33% in the FTLD group using the same cut-off of ≥6 spots. If more than 6 spots would be used as a cut-off, than we would obtain a higher specificity with a loss of sensitivity.

### Posttranslational Modifications of the Serpin A1 Isoforms

To further characterize the additional Serpin A1 spots, a mass-spectrometric analysis of the isoforms detected in the immunoblots was done by LC-MS/MS using a LTQ Orbitrap XL mass-spectrometer. Here, Serpin A1 was detected in all 7 spots from a representative gel of a PDD-patient being the dominant protein in spots 1 through 5 (Figure 2B). Serpin A1 was also detected in spots 6 and 7 but the dominant protein was identified as GC-vitamin D-binding protein precursor.

**Figure 4:**
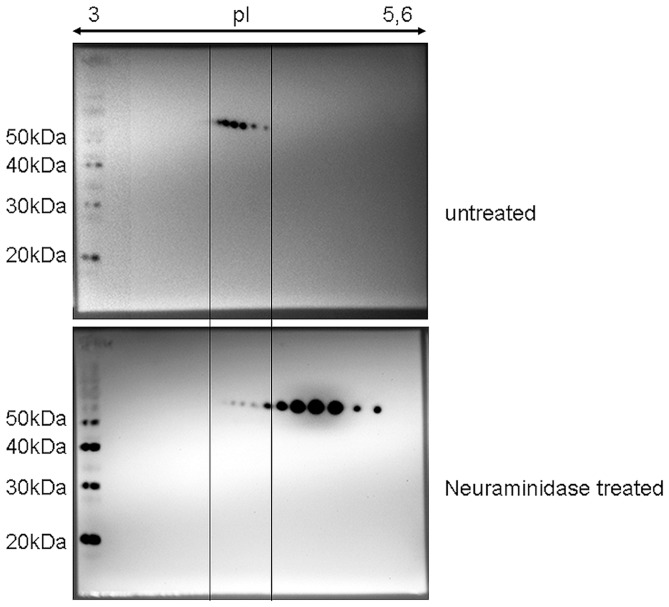
Investigation of posttranslational modifications. Immunoblot of Serpin A1 in a PDD-patient with and without neuraminidase-treatment (different exposures of times are shown to better visualize the individual spots, intensity of both blots were similar; exposure time for the immunoblot without neuraminidase-treatment  = 2 seconds, exposure time for the immunoblot with neuraminidase-treatment  = 10 seconds). Treatment with the enzyme leads to an isoform shift towards a more basic pI and thereby to the disappearance of the diagnostic relevant most acidic spots 1 and/or 2. “Untreated” means usage of a native CSF-sample without neuraminidase-digest.

The analysis of posttranslational modifications with emphasis on possible glycosylations and phosphorylations was performed for the Serpin A1 isoforms. While we failed to identify phosphorylations in any of the Serpin A1-spots, glycosylations were detected for spots 3 to 7 but not for spots 1 and 2 (Table 3) which are the diagnostic relevant ones to differentiate between PD and PDD. As this does not necessarily mean that there are no glycosylations in those spots, a PNGase F digest was performed which revealed that all Serpin A1 spots in a PDD-patient harbour N-glycosylations (Figure S1). However, as the additional Serpin A1 spots are still present after PNGase F treatment, N-linked glycans (or more precisely their terminal sialic acids) cannot be responsible for the altered charge states. We therefore hypothesized that sialylated O-linked glycans may be the underlying posttranslational modifications for the characteristic Serpin A1 spot pattern and tested this hypothesis by performing a neuraminidase-digest. Indeed, we found a shift of the Serpin A1 isoforms towards a more basic pI (Figure 4). Most importantly, the diagnostic relevant acidic spots disappeared, indicating a hypersialylation of those isoforms. This hypersialylation is not due to a decrease in activity of neuraminidase (the enzyme responsible for desialylation), as this was found to be unchanged in CSF (data not shown).

**Table 3:** Posttranslational modifications of Serpin A1.

spot #	m/z (z)	neutral mass (Da)	glycan fragments observed
spot 1	–	–	None observed
spot 2	–	–	None observed
spot 3	906.4291 (+2)	1810.8436	(HexNAc)(Hex)(NeuAc)
spot 4	906.4286 (+2)	1810.8426	(HexNAc)(Hex)(NeuAc)
	670.9987 (+3)	2009.9746	(HexNAc)(Hex)(NeuAc)
	675.6698 (+3)	2023.9900	(HexNAc)(Hex)(NeuAc)
	913.4363 (+2)	1824.8580	(HexNAc)(Hex)(NeuAc)
spot 5	906.4276 (+2)	1810.8406	(HexNAc)(Hex)(NeuAc)
	675.6693 (+3)	2023.9861	(HexNAc)(Hex)(NeuAc)
	670.9987 (+2)	2009.9743	(HexNAc)(Hex)(NeuAc)
	913.4363 (+3)	1824.8580	(HexNAc)(Hex)(NeuAc)
	1223.8602 (+3)	3668.5582	(HexNAc)2(Hex)2(NeuAc)2
	1227.1494 (+3)	3678.4252	(HexNAc)2(Hex)2(NeuAc)2
	1320.8926 (+3)	3959.6572	(HexNAc)2(Hex)2(NeuAc)2
spot 6	760.8801 (+2)	1519.7456	(HexNAc)(Hex)
	778.8799 (+2)	1555.7452	(HexNAc)(Hex)
	906.4281 (+2)	1810.8380	(HexNAc)(Hex)(NeuAc)
	583.3101 (+3)	1746.9085	(HexNAc)(Hex)
	1005.9940 (+2)	2009.9734	(HexNAc)(Hex)(NeuAc)
	1013.0013 (+2)	2023.9884	(HexNAc)(Hex)(NeuAc)
	1020.0094 (+2)	2038.0034	(HexNAc)(Hex)(NeuAc)
	1133.5909 (+2)	2265.1674	(HexNAc)(Hex)(NeuAc)
spot 7	760.8811 (+2)	1519.7476	(HexNAc)(Hex)
	860.4453 (+2)	1718.8784	(HexNAc)(Hex)
	659.0319 (+3)	1974.0739	(HexNAc)(Hex)
	906.4280 (+2)	1810.8414	(HexNAc)(Hex)(NeuAc)
	913.4352 (+2)	1824.8562	(HexNAc)(Hex)(NeuAc)
	1005.9948 (+2)	2009.9734	(HexNAc)(Hex)(NeuAc)
	1013.0012 (+2)	2023.9874	(HexNAc)(Hex)(NeuAc)
	1048.4598 (+4)	4189.8109	(HexNAc)2(Hex)2(NeuAc)2
	1003.0392 (+5)	5010.1586	Indeterminate

Listing of glycosylation residues for Serpin A1 isoforms represented by spot 1 to 7 of a 2D DIGE experiment. Interestingly, spots number 1 and 2 seem not to be glycosylated. Abbreviations: HexNAc  =  N-acetyl-hexosamine, Hex  =  hexose (mannose, glucose or galactose), NeuAc  =  sialinic acid.

### Source of Additional Serpin A1 is the Brain

**Figure 5:**
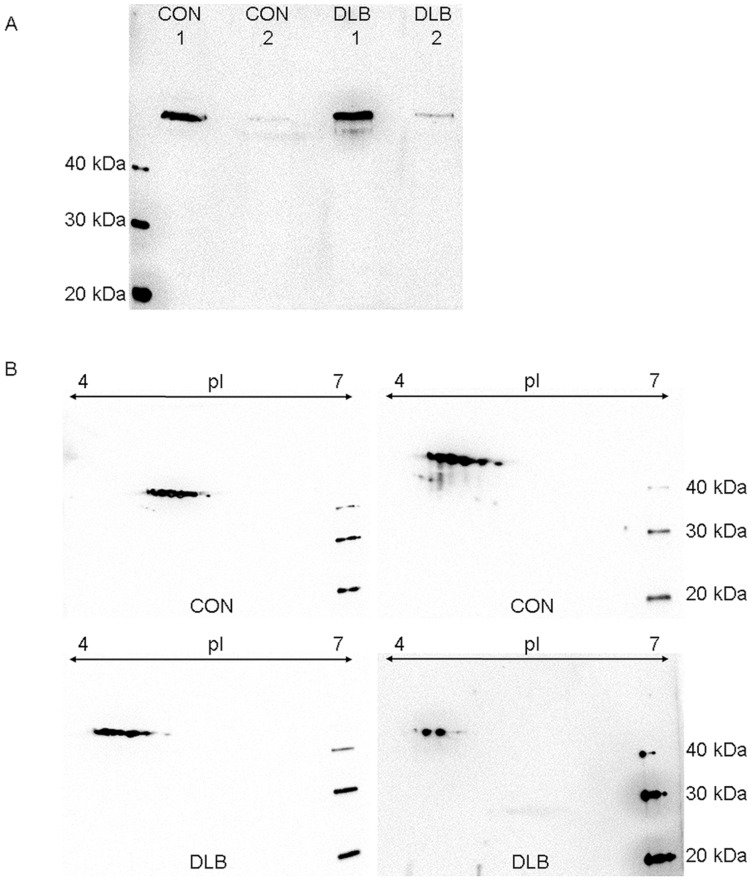
Immunoblots of Serpin A1 in human cortex tissue. **5A** shows 1D-immunoblot in two CON and two patients with Lewy body dementia as a pathophysiological correlate of Parkinson’s dementia. The protein can be identified in both tissues of control persons and PDD patients. **5B** illustrates 2D-immunoblot for Serpin A1 of the patients investigated in 4A. The isoform pattern seen in CSF of CON/PD and PDD (Figure 3C) with spot 1 and/or 2 indicative for PDD could not be reproduced in human cortex tissue. Abbreviations: CON  =  control persons, DLB  =  Lewy body dementia, pI  =  isoelectric point of the proteins.

We analyzed Serpin A1 expression in brain tissues to investigate whether the CSF protein amounts can be traced back to protein release from the brain tissue into the CSF. As our patients show no elevation of the age-dependent elevated Q-Alb (representation of the blood-CSF barrier function), flow from the blood into the CSF as source of our results is not likely. Nevertheless, we can not rule out, that the Serpin A1 amount in the CSF has its source in the choroid plexus with consecutive release into the CSF. 1D- and 2D-immunoblot analysis revealed Serpin A1 expression in brain material from both CON and patients with Lewy body dementia which represent a pathologic pendant for PDD (Figure 5A). However, the additional isoforms of Serpin A1 were not restriced to patients with DLB and can also be identified in CON (Figure 5B).

To investigate if the additional Serpin A1 spots were a direct result of cell destruction in the brain, we correlated tau-values above 450 pg/ml and the number of Serpin A1 spots ≥6 performing Spearman-rank correlations. No significant correlation was found in the various subgroups (PD: r = −0.102, p = 0.663; PDD: r = 0.428, p = 0.0584; AD: r = 0.169, p = 0.662; FTLD: r = 0.0, p = 1.0), so that we suppose that the Serpin A1 isoform-distribution is a tau-level independent marker for PDD. We intentionally did not investigate or compare values of amyloid ß 1–42 because of the decreased stability of the protein. Our intention was to avoid the preanalytical bias of false Aß 1–42 measurement when comparing our samples to those from Perugia or Kuopio.

## Discussion

Parkinson’s disease dementia is diagnosed according to clinical criteria and neuropsychological examinations [27], [28]. Since the typical Parkinson motor-symptoms are initially predominant, the cognitive impairments or even a dementia is often neglected and detected in advanced stages [29]. In order to identify PD patients who are at risk to develop a dementia, a laboratory biomarker would be of great advantage.

In this study, CSF-analysis of patients with PD and PDD was performed using proteomic methods in order to detect proteins of potential diagnostic value. Of the six proteins identified, only the serine-protease-inhibitor Serpin A1 could be verified with biochemical methods to be statistically significant regulated – a protein that was already described to be relevant in AD and DLB [30]. However in the validation phase, there was a large overlap among the different groups investigated so that according to those experiments the total level of Serpin A1 can not be used as a diagnostic marker. Therefore, we characterized the isoform-distribution of Serpin A1 and identified a different protein-pattern with 6 or more spots in PDD and with 5 spots or less in PD and CON where spots 1 and/or 2 were indicative for differentiation of PDD with 100% sensitivity and 58% specificity. These results could be verified in a larger patient-cohort of three different specialized centers (Ulm/Germany, Perugia/Italy and Kuopio/Finland) whereby two PD cases with a 7/9 spot-pattern interestingly developed a dementia in the course of their disease. This may be suggestive that the isoform-pattern does not only help to support the diagnosis but has also a predictive value, a hypothesis that has to be followed up in further studies.

Although our principal goal was the identification of a biomarker that differentiates PD patients from those with additional dementia at the time of lumbar puncture in a cohort with clinically and neuropsychologically proven diagnoses, we additionally investigated the 2D spot pattern in other dementia diseases to get the specificity of the protein among dementia-subgroups. Here, patients with AD revealed in some cases (specificity 71%) and in FTLD in more cases the typical spot pattern (specificity 33%). Nevertheless, statistic based conclusions can not be stated because of the small number of patients in the subgroups and has to be reproduced with larger patient cohorts.

2D-immunoblotting is a sophisticated method and not applicable for routine assessments, so that further analyses of posttranslational modifications were performed and provided evidence for glycosylation of the Serpin A1-isoforms. To investigate Serpin A1 glycosylation in more detail, we used the enzymes PNGase F to remove N-linked glycans as well as neuraminidase to remove terminal sialylation from N- and O-linked glycans. Only treatment with the latter resulted in a shift of the Serpin A1 isoforms towards a more basic pI and thereby in the disappearance of the two relevant most acidic spots in PDD, indicating an O-linked hypersialylation to be responsible for altered charged states of those isoforms. However, this hypersialylation is obviously not caused by an impaired neuraminidase function, as activity of this enzyme does not change in the CSF of any of the groups analysed. This may be the basis for future promising approaches to establish a routine diagnostic assay for the diagnosis/differential diagnosis of PD/PDD.

Since Serpin A1 belongs to the acute-phase proteins [31] in plasma and it is known to be expressed in the liver and also in macrophages [32], we had to confirm that Serpin A1 was indeed brain derived. In a pilot experiment, human cortex samples of patients with DLB and CON were analysed for Serpin A1 expression that could be investigated in brain tissue of both diseased patients and CON without difference in the protein-spot pattern. On the basis of these results, we supposed that the additionally and hypersialylated isoforms in PDD are released into the CSF, an assumption that is indirectly supported by the previous finding that unglycosylated Serpin A1 or Serpin A1 with reduced glycosylation is not secreted into the blood by hepatocytes [33]. Assuming that this is also true for neurons, it would explain why Serpin A1 can not differentiate between DLB and CON in brain tissue. To rule out that the Serpin A1 isoforms are a pathophysiological correlate of general cell destruction, we performed correlation analyses with tau protein without differences of both proteins in our groups.

In general, glycosylation events have already been implicated in the pathogenesis of neurodegenerative diseases [34], [35], [36]. However for PD and PDD, only little is known about the relevant pathomechanisms of glycosylations and sialylations whereby a role of alpha-synuclein glycosylation is discussed in the formation of protein inclusions and disease progression [37], [38]. Wang et al. found that the phosphorylated alpha-synuclein is able to distinguish between PD and atypical parkinsonian syndroms like multisystem atrophy or supranuclear palsy. This may indicate that investigation of posttranslational modification of proteins is a better marker to distinguish diseases than the proteins itsself [39]. Analogous to AD, Huntington disease and CJD [40], an accumulation of pathological aggregates through serine-protease-inhibitors – in addition to the role of alpha-synuclein in the pathogenesis of the disease [41], [42], [43] - can be assumed and PDD could therefore be a subgroup of neurodegenerative diseases with cerebral protein aggregation.

Interestingly, Serpin A1 is not only involved in folding of other proteins but also is, like tau protein and amyloid-beta peptides, able to polymerise and form aggregates itself [44], [45], [46]. Those aggregates were investigated in some diseases with liver-cirrhosis where Serpin A1-aggregation can be detected in liver tissue [45]. Additionally, those aggregations were found to be relevant in the development of a dementia syndrome caused by autosomal-dominant familiar encephalopathy with neuroserpin inclusion bodies, indicating that both diseases may belong to the common disease entity of serpinopathies [45], [47], [48]. One could hypothesize that the formation of Serpin A1 aggregates takes place in PDD, possibly triggered by differences in posttranslational modifications – (hyper-)sialylation instead of phosphorylation – leading to a different structure and (mal-)function of the protein so that the formation of aggregates is favoured. In order to refute or confirm this theory, further investigations especially pathophysiological-, histological- and animal-based ones are necessary. Here, a comprehensive amount of patients should be investigated.

Independent of these pathophysiological hypotheses, we suppose that the hypersialylated isoforms of Serpin A1 have a predictive value for the development of dementia in PD patients which is worth to be followed up.

## Materials and Methods

### Ethics Statement

This study was conducted according to the principles expressed in the Declaration of Helsinki. The local ethics committees (Ethik-Kommission der Medizinischen Fakultät der Universität Ulm, approval numbers: 8801 and 100305 and the regional Ethical Committee Board (CEAS) of the University of Perugia, protocol number 19369/08/AV as well as the Ethics Committee of Kuopio University Hospital, number 5/2002) approved all experiments within our study. All patients provided written informed consent for the collection of samples and subsequent analysis. In case of severe demented patients, their relatives gave written informed consent to their participation in the study. The capacitiy of the patients to consent was assessed by means of clinical, neurological and neuroradiological examinations as well as a neuropsychological screening to investigate global cognitive functions. All PD and PDD patients underwent a detailed psychometric test battery (in detail described in [26]), covering the following tests: MMSE, Geriatric Depression scale, Parkinson Neuropsychometric Dementia Assessment, Regensburger Wortfluessigkeitstest (RWT), Doors Test, Alertness/Go/NoGo/geteilte Aufmerkamkeit, Boston Naming Test, Wechsler Memory Scale (WMS-R), Melmstaedter, Coloured Progressive Marices, VOSP, Clock Test.

### Patients

All CSF samples used for the proteomic approach were taken from patients attending the general outpatient clinic (University of Ulm, Department of Neurology) in 2006/2007. CSF was stored at −80°C after analysis of the routine parameters cell count, lactate, Q-albumin and total protein until further analysis. For the validation study, additional samples were obtained in blinded manner from two different centers: Department of Neurology, Kuopio, Finland (9 PD, 7 PDD) and Department of Neurology, Perugia, Italy (8 PD, 8 PDD).

All individuals underwent a clinical, neurological, neuroradiological examination and a short neuropsychological screening to investigate global cognitive functioning. Patients were examined neuropsychologically for unambiguous classification of their mental status and exclusion of depressive syndroms. All PD and PDD patients in Ulm were investigated with a detailed psychometric test battery, described in [28].

### Patients with PD, PDD and other Dementia (AD and FTLD)

The number of patients per group, tau protein values, and the minimental status test (MMST) for all groups and Hoehn&Yahr stages for PD and PDD are indicated in Table 1. Tau protein was measured in each clinical center.

The diagnosis of all PD/PDD patients was made in accordance with the consensus criteria for PD/PDD [13] as well as on the basis of the DSM-IV criteria and was established by neurologists and neuro-psychologists, both blinded with regard to the neurochemical outcome measures. Patients with PD were on medication with L-dopa agonists or on L-dopa itsself; patients with PDD were additionally treated with rivastigmine.

Diagnosis of Alzheimer’s disease (AD) was set according to the NINCDS-ADRDA criteria [49], the appropriate diagnosis of FTLD-patients was done in accordance with the consensus criteria for FTLD [50], [51] as well as on the basis of the DSM-IV criteria.

### Control Subjects (CON)

The control patients showed neither extrapyramidal-motor nor dementia-specific symptoms. The final diagnoses of the patients were as follows: vertigo (n = 2), paresthesia (n = 2), ischemia (n = 4), complex focal seizures (n = 3), pseudotumor cerebri (n = 1), lumbo-ischialgia (n = 3), migraine (n = 1), sharp-syndrom (n = 1), Tolosa Hunt syndrom (n = 1), Arteriitis temporalis (n = 2), polyradiculopathy (n = 1), transient global amnesia (n = 2) and dissociative disorders (n = 1).

### Neuropathology

Samples of human brain cortex tissues from 2 patients with PDD/DLB (age of 63/80 years, tau-pathology of Braak stage II and III, Lewy-bodies neocortically localized) and 2 CON (age of 59/46 years, tau-pathology of Braak stage 0 and I, no Lewy-bodies) were obtained from the German Brain Bank (Ludwig-Maximilians University, Munich). PDD is neuropathologically characterized by cortical Lewy bodies that also occur in patients with dementia with Lewy bodies. However it is heretofore unclear whether both diseases are a matter of a single one.

### CyDye Labeling

Proteomic analysis via 2D-DIGE was done with a volume-based normalization as described previously [26] with the exception that 6 individual samples of each group were compared. In brief, 400 µl of each CSF sample were concentrated by VivaSpin columns with a 3 kDa cut-off (Sartorius Biolabs products), then albumin and immunglobuline were depleted. For conventional gel staining, the depleted CSF was acetone-precipitated and resuspended in 7 M urea, 2 M thiourea, 4% CHAPS, 1% DTT, 1% IPG Buffer (40%) pH 4–7 by rocking for 1 h at ambient temperature. For CyDye labeling, precipitated proteins were lysed in 7 M Urea, 2 M Thiourea, 4% CHAPS, 30 mM Tris-HCl pH 8.1 at 10°C. Insoluble fractions were removed by centrifugation. For CSF proteome comparison in the first instance, 6 individual CSF samples of each group were compared by the mixed internal standard methodology described by Alban et al. [52]. CSF proteins were labeled with CyDyes™ (GE Healthcare), fluorescent dyes developed for the difference gel electrophoresis-system. Individual samples were labeled either with Cy3 or Cy5 for a dye-switched comparison to avoid potential dye-to-protein preferences. For the mixed internal standard, aliquots of each individual sample included in the experiment were pooled and labeled with Cy2 in the same dye-to-CSF ratio. The labeling reaction was stopped by 20 nmol lysine. The labeled samples were combined and diluted 1.33 x by a stock solution containing 7 M urea, 2 M thiourea, 4% CHAPS, 4% IPG-buffer pH 4–7, 4% DTT w/v for subsequent IEF.

### 2D Gel Electrophoresis and Imaging

Isoelectric focusing was done as described previously [26]. Second dimension SDS-PAGE was performed with homogeneous 12.5% gels (254×200 mm) according to Tastet et al. [53] at 3.5 W/gel overnight at 20°C. The fluorescence signals of the 3 differently Cy-labeled protein samples were imaged using a laser scanner (DIGE Imager, GE Healthcare) recording emission wavelengths of 520 nm (Cy2), 580 nm (Cy3) and 670 nm (Cy5). Proteins were post-stained with silver. Spots of interest were excised manually and subjected to mass spectrometric protein identification.

### In-gel Digest, Mass Spectrometry and Database Search

Manually excised gel plugs were subjected to an automated platform for the identification of gel-separated proteins [54] as described in recent DIGE-based [26], [55], [56] and large-scale proteome studies [56], [57]. Briefly, a peptide mass fingerprint (PMF) and six fragment ion spectra for each sample were recorded automatically with an Ultraflex MALDI-ToF mass spectrometer (Bruker Daltonics) under the control of the FlexControl 3.0 operation software. Post-processing of mass spectra and generation of peak lists was performed with the FlexAnalysis 3.0 software (Bruker Daltonics).

PMF and MS/MS data sets were batch-processed using the BioTools 3.1 software (Bruker Daltonics) as interface to the Mascot 2.2 software (Matrix Science) licensed in-house. Database searches were performed in the Swiss-Prot primary sequence database, restricted to the taxonomy *homo sapiens.* Carboxamidomethylation of Cys was specified as fixed and oxidation of Met as variable modification. One trypsin missed cleavage was allowed. Mass tolerances were set to 100 ppm for PMF searches and to 100 ppm (precursor ions) and 0.7 Da (fragment ions) for MS/MS ion searches. The minimal requirement for accepting a protein as identified was at least one peptide sequence match above identity threshold in coincidence with at least 20% sequence coverage assigned in the PMF.

### Characterization of Serpin A1 Isoforms LC-MS/MS

Samples were subjected to proteolytic digestion on a ProGest (Genomic Solutions) workstation as follows: Samples were reduced with DTT at 60°C and then cooled to room temperature. Furthermore, samples were alkylated with iodoacetamide and subsequently incubated at 37°C for 4 h in the presence of trypsin. Formic acid was added to stop the reaction and the supernatant was analyzed directly by nano LC/MS/MS on a ThermoFisher LTQ Orbitrap XL. 30 µl of hydrolysate was loaded onto a 5 mm×75 µm ID C12 (Jupiter Proteo, Phenomenex) vented column at a flow-rate of 10 µL/min. Gradient elution was over a 15 cm×75 µm ID C12 column at 300 nL/min. A 30 min gradient was employed. The mass spectrometer was operated in data-dependent mode and the six most abundant ions were selected for MS/MS. The Orbitrap MS scan was performed at 60,000 FWHM resolutions. MS/MS data were searched using a local copy of Mascot (www.matrixscience.com). The parameters for all LC/MS/MS searches were as follows: Type of search: MS/MS ion search. Taxonomy: human. Enzyme: trypsin. Fixed modifications: carbamidomethyl (C). Variable modifications: oxidation (M), acetyl (N-term), pyro-glu (N-term Q), methyl (various), deamidation (NQ), PO4 (STY). Mass values: monoisotopic. Protein mass: unrestricted. Peptide mass tolerance: ±10 ppm (Orbitrap). Fragment mass tolerance: ±0.5 Da (LTQ). Maximum missed cleavages: 2.

### Immunoblotting

Equal amounts of total protein or equal volumes (CSF) were denatured and subjected to a SDS-PAGE in 12% polyacrylamide gels. Proteins were transferred onto PVDF membranes (Millipore, USA), correct transfer was checked by Ponceau Red S staining. The membranes were incubated with the respective primary antibody (see below) followed by incubation with HRP conjugated secondary antibodies. Signal detection was performed by enhanced chemiluminescence (GE healthcare) on a CCD-camera. For 2D-immunoblotting, strips were equilibrated for 2×20 min in 6 M urea, 125 mM Tris-HCL pH 7.85, 3% SDS and 20% glycerol (v/v). 1% dithiothreitol (DTT) and 4.2% iodoacetic acid (IAA) were added for the first and second equilibration step, respectively. The following primary antibodies were used: Ceruloplasmin (BD-Biosciences 611488), Fetuin A (R&D Systems BAF1184), Haptoglobin Hp2 (Abcam AB52652), Serpins A1, A8, F1 (R&D Systems MAB1268, BAF3156, BAF 1177) and Zinc-alpha-2 Glycoprotein (BD Biosciences 612354).

### PNGase F and Neuraminidase Digests

In order to assess possible glycosylations or sialylations of the serpin A1 isoforms, 5 µl of CSF was digested with PNGase F (New England Biolabs) or neuraminidase (Roche, 1585886) as stated by the manufacturers and subjected to a 2D-immunoblot.

### Neuraminidase Assay

To quantitatively assess the neuraminidase level in CSF, a commercially available Neuraminidase Assay kit (Molecular probes) was used according to the manufacturer’s instructions. CSF was measured in a 1+1 dilution.

### Calculations and Statistics

Band volumes of immunoblots (adjusted for membrane background) were determined using the Quantity One software (BioRad).

Analysis for significant differences in a given parameter between all tested groups or between two groups were calculated by Kruskal-Wallis test or Mann-Whitney test. Correlation between parameters was examined applying Spearman rank correlation. P-values p≤0.05 were considered to be significant. For ROC analysis, p-values p≤0.01 were considered significant (sigma plot software 10.0). Standard measures of diagnostic test validity such as sensitivity and specificity were calculated for the diagnostic groups [58].

## Supporting Information

Figure S1 **Representative Serpin A1-blots of PNGase F-treated CSF of PD and PDD.** Abbreviations: PD = Parkinson’s disease, PDD  =  Parkinson’s disease dementia
(TIF)
