# Supplementary figures and images for: Differential Sialylation of Serpin A1 in the Early Diagnosis of Parkinson’s Disease Dementia
Source: PLoS One. 2012 Nov 8;7(11):e48783. doi: 10.1371/journal.pone.0048783 (PMC3493604; doi:10.1371/journal.pone.0048783)

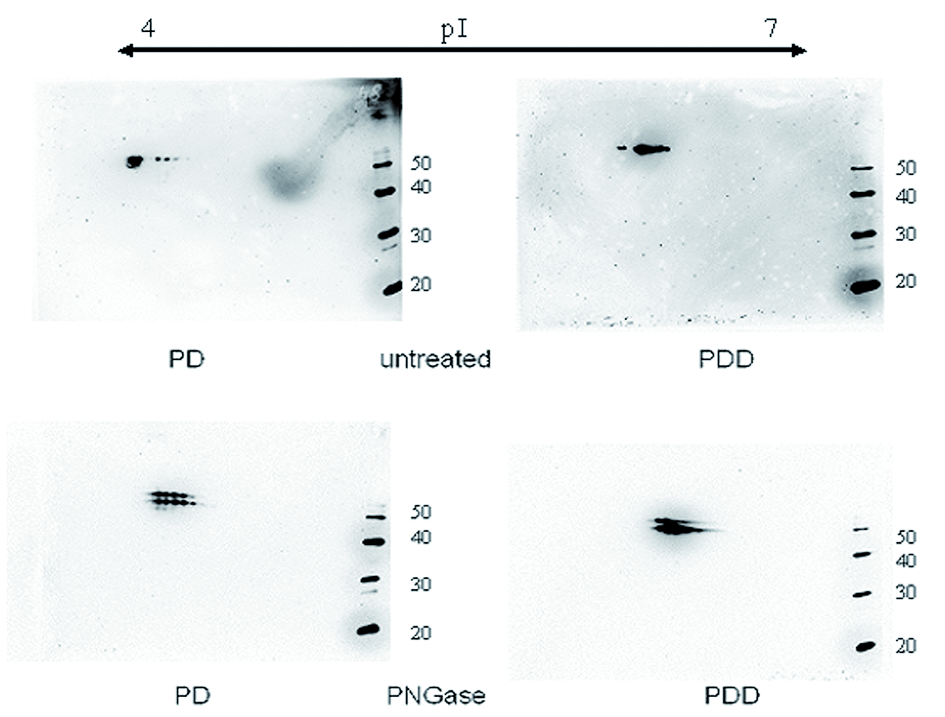

Supplement: Figure S1 — Representative Serpin A1-blots of PNGase F-treated CSF of PD and PDD. Abbreviations: PD = Parkinson’s disease, PDD = Parkinson’s disease dementia (TIF) [file pone.0048783.s001.tif]
